# Case Report: Technetium-99m Pertechnetate Scintigraphy Findings in a Dog With Iodine Deficiency-Induced Goitrous Hypothyroidism

**DOI:** 10.3389/fvets.2022.922456

**Published:** 2022-06-13

**Authors:** Taesik Yun, Yejin Na, Dohee Lee, Yoonhoi Koo, Yeon Chae, Hyeyeon Nam, Byeong-Teck Kang, Mhan-Pyo Yang, Hakhyun Kim

**Affiliations:** Laboratory of Veterinary Internal Medicine, College of Veterinary Medicine, Chungbuk National University, Cheongju, South Korea

**Keywords:** ^99m^TcO_4_^-^, canine, goiter, home-cooked diet, hypothyroidism, urinary iodine concentration, nuclear medicine, scintigraphy

## Abstract

There is only one previous report of canine goitrous hypothyroidism caused by iodine deficiency from 1986. The present case report describes the novel diagnostic methods and long-term outcomes of a dog diagnosed with goitrous hypothyroidism caused by iodine deficiency. A 4-year-old neutered, female Pomeranian dog presented with a cervical mass, lethargy, and inactivity. The dog had a history of eating home-cooked diets sold by a private seller for 1 year. The physical examination and ultrasonography showed two bilaterally symmetric masses in the mid-cervical area (left, 1.8 × 1.4 cm; right, 2.3 × 1.8 cm), and they were suspected to be the thyroid glands. To identify the function of the thyroid gland, the basal concentrations of thyroid hormones [total T4 (tT4) and thyroid-stimulating hormone (TSH)] were measured and a TSH stimulation test was performed: baseline tT4, 0.5 μg/dL (reference interval, 1–4 μg/dL), baseline TSH, 0.81 μg/dL (reference interval, 0.05–0.42 μg/dL), and post-tT4, 1 μg/dL (6 h after the injection of TSH). The values indicated primary hypothyroidism. The urinary iodine concentration was 302 μg/L, which was markedly lower than that of normal dogs (1,289 μg/L). Thyroid scintigraphy with technetium-99m pertechnetate was also performed to quantify the activity of the thyroid gland, and the thyroid-to-salivary ratio was 3.35. Based on the results of these examinations and patient history, the dog was diagnosed with diet-induced (iodine deficiency) goitrous hypothyroidism. The dog was treated with iodine (62.5 μg/day). At 31 days after treatment, clinical signs and thyroid hormones were normalized (tT4, 1.3 μg/dL; TSH, 0.24 μg/dL). One year after treatment, the dog was well with normal concentrations of thyroid hormones (tT4, 1.8 μg/dL; TSH, 0.27 μg/dL) and a partially reduced goiter (left, 1.6 × 1.1 cm; right, 1.2 × 0.9 cm). This is the first case to describe novel diagnostic methods and long-term outcomes of a dog diagnosed with goitrous hypothyroidism caused by iodine deficiency.

## Introduction

Hypothyroidism is an endocrine disorder in which the thyroid gland cannot secrete sufficient levels of thyroid hormones (1). Among the potential causes of canine hypothyroidism, acquired primary hypothyroidism is the most common type, accounting for >95% of these cases (1, 2). Other rare etiologies of primary hypothyroidism include iodine deficiency, congenital hypothyroidism, ingestion of goitrogen, drug administration, neoplasia, surgical thyroidectomy, and iodine radiotherapy (2).

Goiter is a clinical term for an abnormal enlargement of the thyroid gland. A goiter could be due to hyperthyroidism or hypothyroidism depending on whether the gland secretes too much or too little hormones. In humans, iodine deficiency is the most common cause of a goiter, and it is commonly observed in countries that rarely use iodized salt (3). When thyroid hormones are deficient due to iodine deficiency, which is needed for the production of thyroid hormones, negative feedback to the hypothalamus is reduced, which triggers a compensatory increase in the secretion of the thyrotropin-releasing hormone and thyroid-stimulating hormone (TSH) (4). When the hypothalamus-pituitary-thyroid axis is functioning, this increased secretion can induce hyperplasia and hypertrophy of thyroid follicular cells, resulting in goitrous changes in the thyroid gland.

Thyroid scintigraphy with technetium-99m pertechnetate (^99m^${\text{TcO}}_{4}^{-}$) has been used to evaluate thyroid gland function in veterinary medicine (2, 5). Scintigraphy is considered to be one of the gold standard diagnostic tools for differentiating dogs with hypothyroidism from euthyroid dogs. Primary hypothyroidism shows low accumulation of ^99m^${\text{TcO}}_{4}^{-}$ in the thyroid gland, and the size of the thyroid gland might also appear smaller than that of the normal thyroid gland. However, there are no reports of thyroid scintigraphy in canine goitrous hypothyroidism.

There was only one report of canine goitrous hypothyroidism caused by iodine deficiency in 1986 (6). However, because the report was published so long ago, there is a lack of detailed information, such as the diagnostic methods used and long-term treatment outcomes. Therefore, the purpose of this case report is to describe the novel diagnostic methods and long-term outcomes of a dog diagnosed with goitrous hypothyroidism caused by iodine deficiency.

## Case Presentation

**Figure 1:**
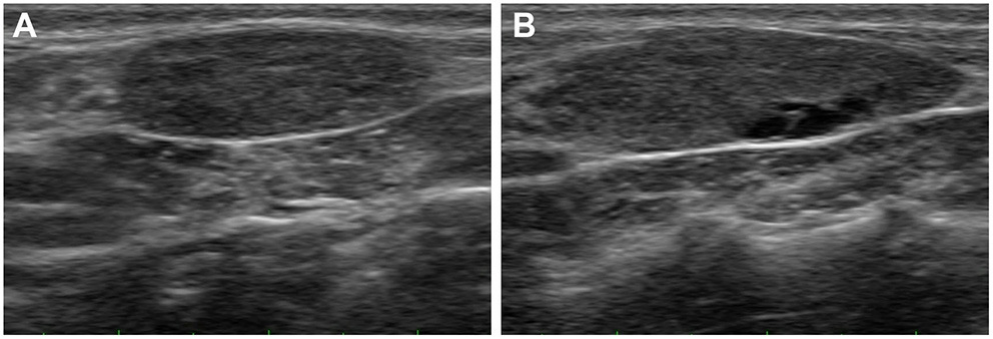
Ultrasonograms of the goiter caused by iodine deficiency in the dog. The echotexture of the left thyroid lobe **(A)** is homogeneous, whereas the echotexture of the right lobe **(B)** is heterogenous with some anechoic lesions. Both thyroid lobes are mildly enlarged and well marginated without invasion.

**Figure 2:**
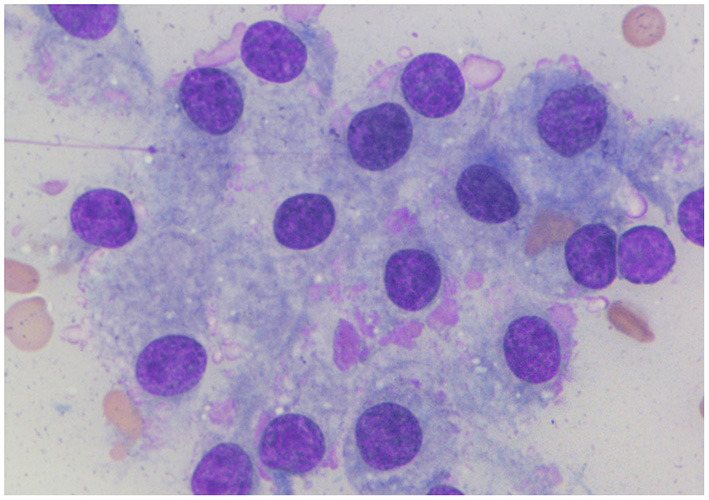
Cytological findings of the goiter caused by iodine deficiency in the dog. Microscopic analysis shows that the cells are polygonal in shape with or without an intercellular border. Nuclei are eccentrically located and have a mild coarse chromatin pattern with basophilic nucleoli. Additionally, there is a low nucleus-to-cytoplasm ratio. Wright-Giemsa.

A 4-year-old neutered, female Pomeranian dog presented with a cervical mass, lethargy, and inactivity. The dog had a history of eating home-cooked diets (non-standardized and non-qualified food) sold by a private seller for 1 year. The physical examination revealed two bilaterally symmetric masses in the mid-cervical area. Ultrasonography was performed to evaluate the masses. The echotexture of the right thyroid lobe was heterogeneous, with some anechoic lesions, whereas the echotexture of the left lobe was homogeneous (Figures 1A,B). Both masses were mildly enlarged (left, 1.8 × 1.4 cm; right, 2.3 × 1.8 cm) and well marginated without invasion. Fine needle aspiration was also performed for cytological evaluation. Microscopic analysis showed that the cells were polygonal in shape, with or without intercellular borders (Figure 2). The nuclei were eccentrically located and showed a mild coarse chromatin pattern with basophilic nucleoli. On the basis of the aforementioned information, the masses were suspected to be enlarged thyroid glands. Additionally, the nucleus-to-cytoplasm ratio was low. Basal concentrations of thyroid hormones [total T4 (tT4) and TSH] were measured to assess thyroid gland function. Abnormal results were shown for tT4 and TSH: 0.5 μg/dL (reference interval, 1–4 μg/dL) and 0.81 μg/dL (reference interval, 0.05–0.42 μg/dL), respectively. Furthermore, a TSH stimulation test was performed, and the post-tT4 concentration (6 h after injection of TSH) was 1 μg/dL, indicating primary hypothyroidism.

The urinary iodine concentration (UIC) was measured to differentiate iodine excess or deficiency as the cause of hypothyroidism. The UIC of this dog was measured by a commercial laboratory (EONE Laboratories, Incheon, South Korea) by inductively coupled plasma-mass spectrometry using a urine sample stored at −80°C immediately after collection; the UIC was 302 μg/L. Additionally, the UIC of normal dogs [*n* = 5; two male dogs, three female dogs; median age, 8.1 years (range, 1.6–12 years); one Shih-Tzu, two Beagles, one Pomeranian, one Pointer] was also measured because the normal range of UIC in healthy dogs has not been clearly identified. The median value of UIC in normal dogs was 1,289 μg/L (range, 827–2,080 μg/L). Therefore, based on these results and the history of home-cooked diets, the dog was tentatively diagnosed with diet-induced (iodine deficiency) goitrous hypothyroidism.

**Figure 3:**
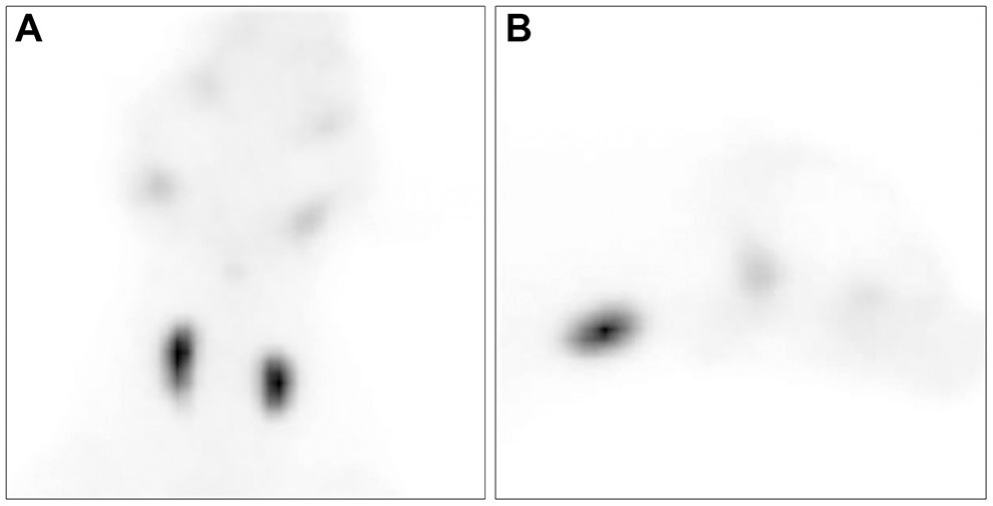
Ventral **(A)** and right lateral **(B)** views of thyroid scintigraphy in the dog with a goiter caused by iodine deficiency. All images were acquired 40 min after the injection of technetium-99m pertechnetate. Markedly increased uptake of technetium-99m pertechnetate is observed in the thyroid mass.

Thyroid scintigraphy using a gamma camera (Dilon 6800 Gamma Camera; Dilon Technologies, Newport News, VA, USA) with ^99m^${\text{TcO}}_{4}^{-}$ was performed to accurately evaluate the size and function of the thyroid gland. ^99m^${\text{TcO}}_{4}^{-}$ (79.2 MBq/kg) was administered intravenously into the right cephalic vein. Scintigraphy scans were obtained 40 min after ^99m^${\text{TcO}}_{4}^{-}$ injection. Dorsal and sagittal images were obtained for 200,000 counts. The acquired images were processed using a dedicated computer running program (Dilon 6800 software). Remarkable uptake of ^99m^${\text{TcO}}_{4}^{-}$ was observed in both thyroid glands (Figures 3A,B). The sizes of the oval-shaped thyroid masses were 2.3 × 1.6 cm (left) and 2.7 × 1.7 cm (right). To quantify thyroid activity, the thyroid-to-salivary (TS) ratio was calculated to be 3.35.

Because the owner refused to change to a nutritionally balanced commercial diet, the dog was treated with iodine supplementation (62.5 μg/day; Kelp, NOW Foods, Bloomingdale, IL, USA) based on the dose recommended by the Association of American Feed Control Officials methods for substantiating the nutritional adequacy of dog and cat foods (7). At 31 days after iodine supplementation, the concentrations of tT4 and TSH were measured to evaluate the function of the thyroid gland, and they were all normalized (tT4, 1.3 μg/dL; TSH, 0.24 μg/dL). However, the thyroid gland size remained unchanged. At 192 days after iodine supplementation, the concentrations of tT4 and TSH were 1.1 μg/dL and <0.1 μg/dL, respectively. Although the thyroid gland could still be palpated, its size was partially reduced (left, 1.6 × 1.1 cm; right, 1.2 × 1.0 cm). One year after iodine supplementation, the dog was well with the normal concentrations of thyroid hormones (tT4, 1.8 μg/dL; TSH, 0.27 μg/dL) despite a partially reduced goitrous mass (left, 1.6 × 1.1 cm; right, 1.2 × 0.9 cm).

## Discussion

This case report described the clinical features of a dog with diet-induced goitrous hypothyroidism. Although the size of the thyroid gland was partially reduced, the concentration of thyroid hormones remained within the normal range for a year of follow-up after iodine therapy. Furthermore, the thyroid scintigraphy findings of canine goiter were identified for the first time in veterinary medicine. Compared with the typical results of hypothyroidism, goitrous hypothyroidism revealed a markedly increased TS ratio.

Although iodine deficiency is one of the most common causes of human primary hypothyroidism (8), it is a rare cause of canine hypothyroidism due to nutritionally balanced commercial diets. Iodine is essential for thyroid hormone production. Therefore, iodine deficiency impairs the synthesis of thyroid hormones and leads to iodine deficiency disorders, such as goiters, hypothyroidism, toxic nodular goiters (hyperthyroidism), impaired mental function, reduced work productivity, decreased fertility rate, abortion, stillbirth, and congenital anomalies (4). One's iodine status can be evaluated using biomarkers of exposure (UIC) and function (thyroid size and hormone levels) (9). Among them, UIC is a well-accepted sensitive marker that reflects the iodine status in humans. It measures recent iodine intake because 92% of dietary iodine is absorbed and the kidneys excrete >90% of dietary iodine within 24–48 h (10–12). In veterinary medicine, the ranges of UIC in dogs with iodine-deficient hypothyroidism and normal dogs have not been clearly established. Therefore, the exact criteria for diagnosing iodine-deficient hypothyroidism remain unknown. Therefore, iodine-deficient hypothyroidism should be diagnosed by considering the various test results, histories, and therapeutic responses after iodine supplementation. The UIC in the present case (302 μg/L) was markedly lower than the median UICs of normal dogs (our study, 1,289 μg/L; Aicher et al.'s study, >1,000 μg/L) (13). Furthermore, the concentrations of tT4 and TSH normalized after iodine supplementation. Therefore, based on the above results and the history of feeding, the dog was diagnosed with goitrous hypothyroidism caused by iodine deficiency.

The TSH stimulation test is regarded as the gold standard diagnostic tool for evaluating thyroid gland function (14). However, thyroid scintigraphy with ^99m^${\text{TcO}}_{4}^{-}$ has recently been regarded as the most accurate method for differentiating primary hypothyroidism from nonthyroidal illnesses (15). The absorption of iodine in thyrocytes occurs using the sodium-iodide symporter (16). Similarly, the uptake of ^99m^${\text{TcO}}_{4}^{-}$ as an analog of iodine by thyroid cells occurs *via* a mechanism similar to that of iodine. In veterinary medicine, the TS ratio of the euthyroid is ~1.0 (17), whereas the uptake of ^99m^${\text{TcO}}_{4}^{-}$ by thyrocytes in dogs with hypothyroidism is reduced (15). In the present case, the uptake of ^99m^${\text{TcO}}_{4}^{-}$ was markedly increased compared to that in the normal thyroid gland, and the TS ratio was 3.35.

Lymphocytic thyroiditis and idiopathic atrophy are the two typical causes of primary hypothyroidism. The pathogenesis of these two causes is associated with the loss of normal thyroid parenchyma due to infiltration of inflammatory cells (lymphocytic thyroiditis) and replacement by adipose connective tissue (idiopathic atrophy) (18). Contrary to lymphocytic thyroiditis and idiopathic atrophy, an iodine deficiency-induced goiter is characterized by hyperplasia and hypertrophy of normal-functioning thyroid follicular cells due to a compensatory increase in the secretion of thyrotropin-releasing hormone and TSH (4). Although the uptake of ^99m^${\text{TcO}}_{4}^{-}$ might be diminished in typical primary hypothyroidism, it might be increased in cases of an iodine deficiency-induced goiter. The kinetics of iodine uptake by various functions of the human thyroid gland show different conditions (19). Compared to euthyroidism, iodine uptake was higher in hyperthyroidism and lower in hypothyroidism. Additionally, in patients with iodine deficiency, iodine uptake was intermediate between hyperthyroidism and euthyroidism. Similarly, as in a human study (19), the uptake of ^99m^${\text{TcO}}_{4}^{-}$ by an iodine deficiency-induced goiter might have increased markedly due to the increased count and size of normal functioning thyroid follicular cells in the present case.

Complete resolution of the goiter did not occur despite iodine treatment for 1 year in the present case. The size of the goiter due to iodine deficiency is expected to decrease slowly over time as iodine supplementation is provided; however, the increased size of the thyroid gland could be permanent in some goiters (20, 21). The main limitation of this case is that a histopathological evaluation was not performed. Therefore, the presence of thyroid tumors was not completely excluded, but thyroid tumors generally occur unilaterally (2). Furthermore, the incidence of hypothyroidism due to thyroid tumors is very low (~10% of thyroid tumors) because there must be significant loss (>80%) of the parenchyma of the thyroid gland (2). Therefore, based on these facts, in addition to the result of UIC and therapeutic response, we believe hypothyroidism in this case may have been caused by iodine deficiency rather than a thyroid tumor.

Our findings suggest that the measurement of UIC and iodine trials might be considered in dogs with goitrous hypothyroidism if a dog has a history of eating non-standardized or qualified diets. This is the first reported case to describe scintigraphy results in a dog with goitrous hypothyroidism. In the future, this finding of a markedly increased TS ratio may provide valuable diagnostic information for canine goiter, although further studies are needed to establish diagnostic criteria for differentiating between goiters and thyroid tumors.

## Data Availability Statement

The original contributions presented in the study are included in the article/supplementary material, further inquiries can be directed to the corresponding author.

## Ethics Statement

Ethical review and approval was not required for the animal study because the case report is a retrospective evaluation with no active interventional or research component. Written informed consent was obtained from the owners for the participation of their animals in this study.

## Author Contributions

TY, YN, DL, HN, and HK contributed to case management. YK, YC, B-TK, and M-PY were responsible for the scintigraphy evaluation. TY wrote the first draft of the manuscript. YN, DL, YK, YC, HN, B-TK, M-PY, and HK participated in the revision of the manuscript. All authors read, commented on, and approved the final manuscript.

## Funding

This work was supported by the National Research Foundation of Korea (NRF) grant funded by the Korea Government (MSIT) (No. NRF-2021R1F1A1061799).

## Conflict of Interest

The authors declare that the research was conducted in the absence of any commercial or financial relationships that could be construed as a potential conflict of interest.

## Publisher's Note

All claims expressed in this article are solely those of the authors and do not necessarily represent those of their affiliated organizations, or those of the publisher, the editors and the reviewers. Any product that may be evaluated in this article, or claim that may be made by its manufacturer, is not guaranteed or endorsed by the publisher.
